# Extracellular vesicle-shuttled miRNAs as a diagnostic and prognostic biomarker and their potential roles in gallbladder cancer patients

**DOI:** 10.1038/s41598-021-91804-0

**Published:** 2021-06-10

**Authors:** Eijiro Ueta, Koichiro Tsutsumi, Hironari Kato, Hiroshi Matsushita, Hidenori Shiraha, Masakuni Fujii, Kazuyuki Matsumoto, Shigeru Horiguchi, Hiroyuki Okada

**Affiliations:** 1 Department of Gastroenterology and Hepatology, Okayama University Graduate School of Medicine, Dentistry and Pharmaceutical Science, Okayama, Japan; 2 Department of Gastroenterology, Okayama University Hospital, 2-5-1 Shikata-cho, Kita-ku, Okayama-city, Okayama 700-8558 Japan; 3 Department of Internal Medicine, Okayama Saiseikai General Hospital, Okayama, Japan

**Keywords:** Tumour biomarkers, Gall bladder cancer

## Abstract

Circulating microRNAs (miRNAs) in serum extracellular vesicles (EVs) are a promising biomarker in cancer. We aimed to elucidate the serum EVs miRNA biomarkers to identify patients with gallbladder cancer (GBC) and to clarify their potential roles. One hundred nineteen serum EVs from GBC and non-GBC individuals were isolated by pure-EVs-yieldable size-exclusion chromatography, and then were analyzed using a comprehensive miRNAs array and RT-qPCR-based validation. The functional roles of the identified miRNAs were also investigated using GBC cell lines. Serum EVs miR-1246 and miR-451a were significantly upregulated and downregulated, respectively in GBC patients (*P* = 0.005 and *P* = 0.001), in line with their expression levels in cancer tissue according to an in silico analysis. The combination of CEA and CA19-9 with miR-1246 showed the highest diagnostic power (AUC, 0.816; Sensitivity, 72.0%; Specificity, 90.8%), and miR-1246 was an independent prognostic marker of GBC (Hazard ratio, 3.05; *P* = 0.017) according to a Cox proportional hazards model. In vitro, miR-1246 promoted cell proliferation and invasion, while miR-451a inhibited cell proliferation and induced apoptosis with the targeting of MIF, PSMB8 and CDKN2D. Taken together, miR-1246 in serum EVs has potential application as a diagnostic and prognostic marker and miR-451a may be a novel therapeutic target in GBC.

## Introduction

Gallbladder cancer (GBC) is among the most common cancers of the biliary system, and has an aggressive pathophysiology^1^. Based on GLOBOCAN 2018, there are approximately 220,000 cases of GBC per year, including cholangiocarcinoma (CCC) and 165,000 deaths a year in the world^2^. There are distinctive geographic and ethnic variations in incidence, which is low in most Western countries but high in some parts of the world, including India, Chile and Japan^3^. However, increasing global migration makes GBC a worldwide disease. The five-year survival rate has improved to > 90% in stage I, but is still < 30% in stages III and IV^4,5^.

Despite recent advances in imaging modalities, there are still problems in both detecting gallbladder lesions and distinguishing GBC from adenoma and non-neoplastic gallbladder disease, including adenomyomatosis (ADM), xanthogranulomatous cholecystitis (XGC), and cholesterol polyp, which form with a protruded gallbladder lesion or wall thickening. First, GBC patients present no specific symptoms, especially in the early stage. In fact, incidentally-discovered GBC after cholecystectomy still occurred. Second, depending on the size, shape and location of gallbladder lesions and concomitant gallstones, it is often difficult to detect the lesion using abdominal ultrasound sonography, multi-detector computed tomography, or endoscopic ultrasound sonography (EUS), which can be vital for an accurate diagnosis and staging of GBC^6^. Other modalities, including magnetic resonance imaging and positron emission and computed tomography are only useful for the preoperative assessment of GBC, to detect the local involvement, regional lymph node metastasis and distant metastasis^7^. Third, bile juice cytology with endoscopic transpapillary gallbladder drainage (ETGD) is available for distinguishing between benign and malignant gallbladder lesions^8^. However, ETGD placement requires high expertise, is associated with a risk of pancreatitis and cystic duct perforation, and induces patient discomfort. Fourth, tumor markers such as carbohydrate antigen 19-9 (CA19-9) and carcinoembryonic antigen (CEA) levels are affected by cholangitis and obstructive jaundice, and thus show insufficient diagnostic ability^9^.Therefore, a novel target that facilitates a high-precision early diagnosis and intensive treatment, besides curative resection, of GBC is needed.

MicroRNAs (miRNAs) are small noncoding RNA molecules, containing approximately 22 nucleotides. They regulate the expression of specific target genes by binding to the 3′-untranslated regions of mRNAs, and either suppressing translation or facilitating mRNA degradation. These epigenetic mechanisms are associated with various diseases, and the miRNA alterations play critical roles in the initiation and progression of cancer. Furthermore, miRNA-expression profiling of human tumors has identified signatures associated with the diagnosis, stage, progression, prognosis and treatment response^10^.

Most circulating miRNAs in blood exist in small extracellular vesicles (EVs) with a lipid bilayer membrane of 40–150 nm in diameter^11^. These EVs are secreted from cancer cells and normal cells, and are shown to contribute to intercellular communications in normal physiological processes and in the pathogenesis of diseases including cancer, in which EVs would transport and transduce miRNAs into target cells, either close or distant from the cells of EV origin through blood circulation. Consequently, EVs-derived miRNAs alter the gene expression and functions of recipient cells^12,13^. Thus, identification of the miRNA profiles in serum EVs will help elucidate the functions of miRNAs in cancer progression and establish novel diagnostic approaches and innovative therapeutics for cancer. However, the specific miRNA profiles of GBC remain to be clarified.

We constructed miRNA expression signatures of GBC and non-GBC using 119 serum EV samples isolated by size-exclusion chromatography (SEC) which was a simple and reproducible method for high-purity isolation from human blood wherein EVs^14^, and used these data to explore miRNAs in serum EVs as diagnostic and prognostic markers. Our samples showed that miR-1246 and miR-451a were significantly upregulated and downregulated in GBC patients, respectively. In addition, the expression levels of these miRNAs in serum EVs were strongly associated with those in tissue (cancer and adjacent normal tissue), indicating the potential application of miR-1246 as a diagnostic and independent prognostic biomarker in GBC. Furthermore, the unknown functional significance of miR-1246 and miR-451a in GBC was investigated. Consequently, we found that miR-1246 promoted proliferation and invasion of GBC cells and miR-451a inhibited cell proliferation and induced apoptosis, strongly suggesting that miR-451a represents a potential therapeutic target in GBC.

## Results

**Figure 1:**
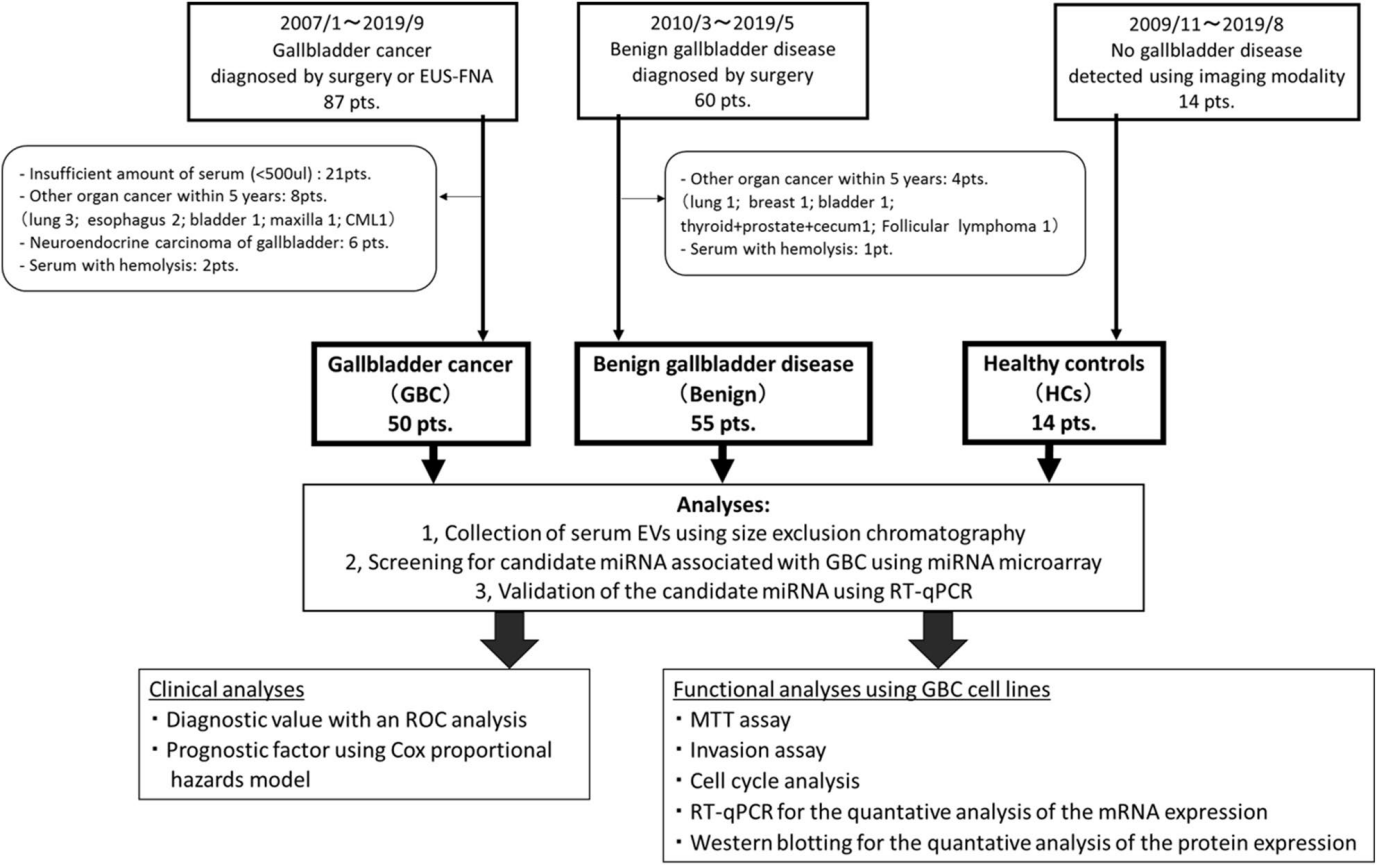
Flowchart of the present study. EUS-FNA, endoscopic ultrasonography-guided fine needle aspiration; CML, chronic myelogenous leukemia; EVs, extracellular vesicles; miRNAs, microRNAs; ROC. Receiver Operating Characteristic; Pts, patients.

A schematic illustration of this study is shown in Fig. 1, and the subject characteristics are shown in Table 1.

**Table 1:** Patient Characteristics.

	Gallbladder cancer	Benign gallbladder disease	Healthy controls
	GBC	Benign	HCs
	N = 50	N = 55	N = 14
Male, n (%)	24 (48)	25 (45)	7 (50)
Age, Median (IQR)	72 (64–78)	63 (50–72)	66 (35–77)
Histology, n	Adenocarcinoma 48	Chronic cholecystitis 35	N.A
	Adenosquamous carcinoma 2	Adenomyomatosis 9	
		Hyperplastic or cholesterol polyp 6	
		Gallstone 6	
		Xanthogranulomatous cholecystisis 2	
		Adenoma 2	
Stage (UICC), n	0, 1; Ia, 1; Ib, 1;	N.A	N.A
	IIa, 3; IIb, 2;		
	IIIa, 6; IIIb, 8;		
	IVa, 4; IVb, 24		
**TNM classification, n**			
N factor	0/1/2 = 18/15/17	N.A	N.A
M factor	0/1 = 34/16	N.A	N.A

UICC, Union for International Cancer Control.

IQR, Interquartile range; N.A., not applicable.

### Characterization of serum EVs isolated by size exclusion chromatography

**Figure 2:**
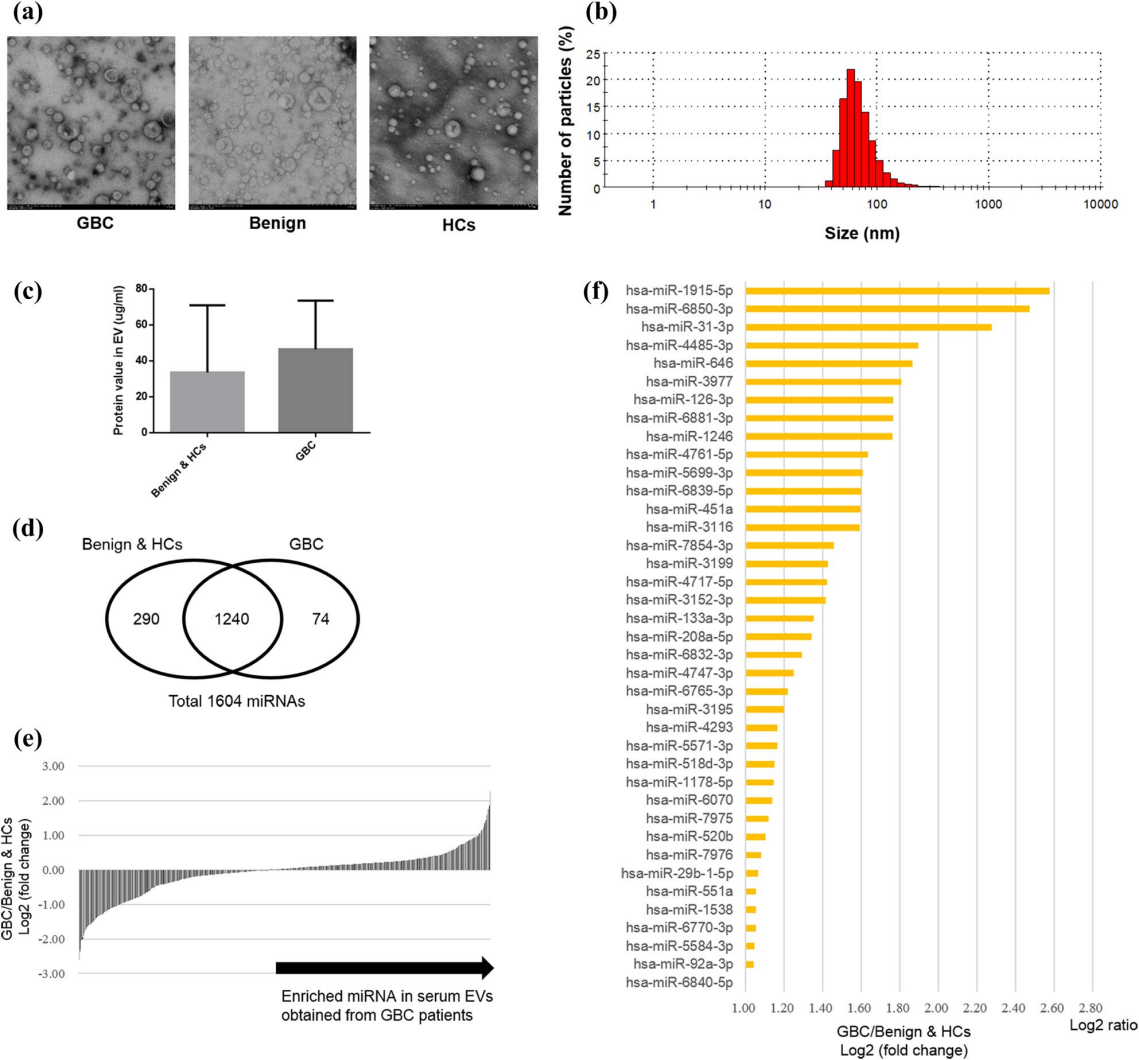
MicroRNA profiling of serum EVs obtained from the patients with gallbladder cancer and benign gallbladder disease, and healthy controls. (**a**) Serum EVs eluted in fraction #4 are observed as particles of 40–150 nm in size by transmission electron microscopy in all groups (× 25,000). Representative images of each group are presented. Scale bars represent 100 nm. GBC, gallbladder cancer; Benign, benign gallbladder disease; HCs, healthy controls. (**b**) The median size of isolated EVs measured by Zetasizer was 72 nm (range: 40–150). A representative image is shown. (**c**) The median protein concentration in #4 fraction was 46.6 μg/ml (IQR: 12.3–73.6) in the GBC (N = 50), and was 33.7 μg/ml (IQR: 19.4–71.0) in the Benign and the HCs (N = 69) (not significant). (**d**) The numbers of detected miRNAs in serum EVs isolated from the GBC and the combination of the Benign and the HCs is shown in a Venn diagram. (**e**) The fold change in the expression of each common 1240 miRNA in serum EVs in the GBC relative to the Benign and the HCs. (**f**) Thirty-nine miRNAs in serum EVs in the GBC showed a > twofold change relative to the Benign and the HCs.

The morphology and size of serum EVs isolated by SEC were similar among the three groups which consisted of 55 patients with GBC, 50 with benign disease (Benign), and 14 healthy controls (HCs), and the size distribution was approximately 40–150 nm in diameter (Fig. 2a,b). The median protein concentration in #4 fraction was higher in the GBC than in the combination of the Benign and the HCs (non-significant) (Fig. 2c).

### The global miRNA expression analysis of serum EVs in gallbladder cancer patients, benign gallbladder disease controls, and healthy individuals

The initial global miRNA screening included the serum samples from 3 patients with GBC, 3 with Benign and 10 HCs (see Supplementary Table S1 online). Among detected total 1604 miRNAs, 1240 miRNAs were common between them (Fig. 2d), and 39 miRNAs were identified to be highly expressed (log2 FC > 1) in the GBC in comparison to the Benign and the HCs (Fig. 2e,f). Particularly, four miRNAs (miR-31-3p, miR-1246, miR-451a, and miR-29b-1-5p) were found in previous articles about cancer progression or epithelial–mesenchymal transition (EMT)^15–18^.

### Upregulation of miR-1246 and downregulation of miR-451a in serum EVs and cancer tissue in GBC patients

**Figure 3:**
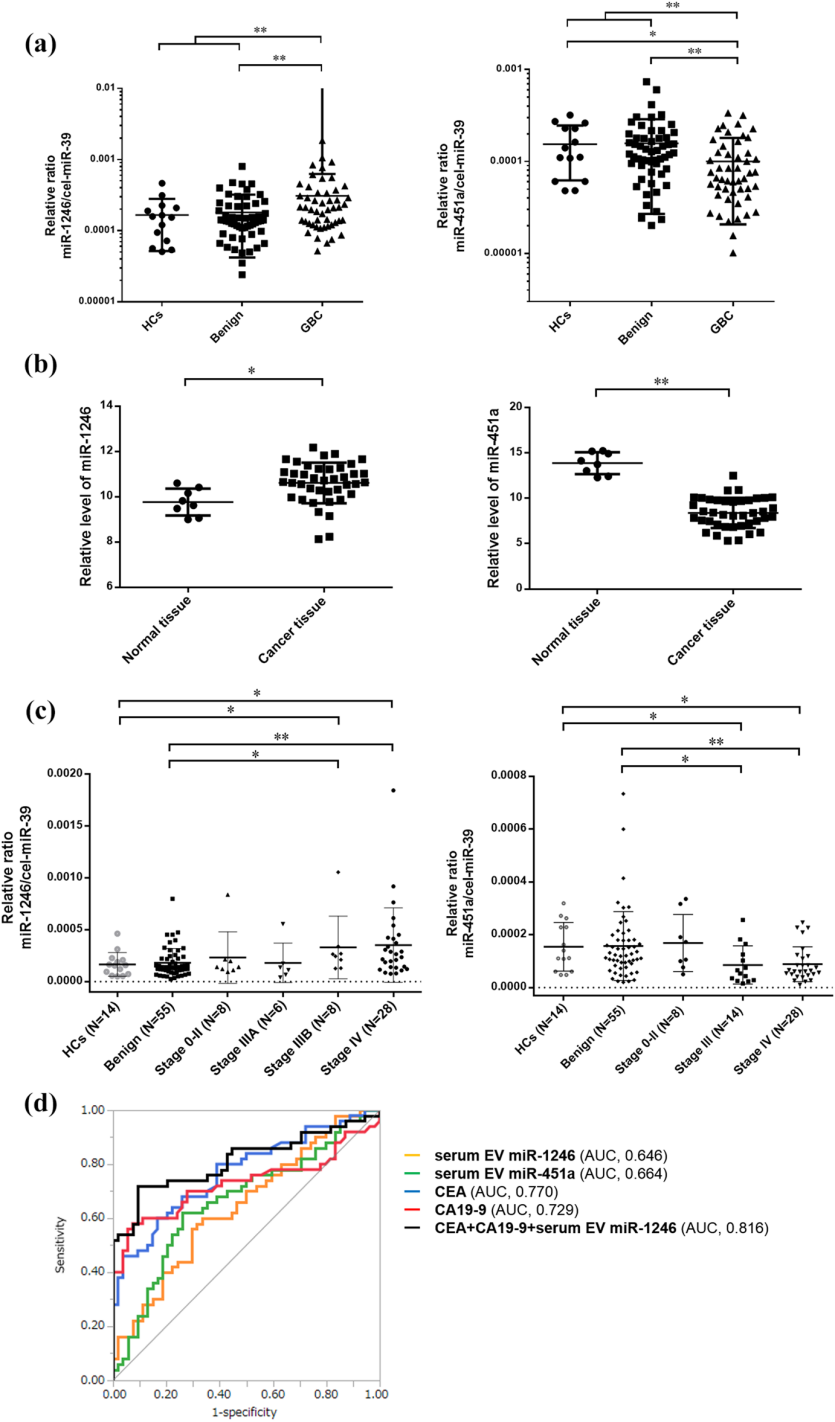
The expression levels of miR-1246 and miR-451a in serum EVs and tissues, and the potential for the application of these miRNAs as diagnostic biomarkers for gallbladder cancer. (**a**) The miR-1246 expression levels in serum EVs in the GBC were significantly higher in comparison to the Benign and the HCs (*P* = 0.005), while the miR-451a expression levels in the GBC were significantly lower in comparison to the Benign and HCs (*P* = 0.001). (**b**) The miR-1246 was significantly upregulated in gallbladder cancer tissues in comparison to normal tissue (FC = 1.79, *P* = 0.029), and the miR-451a was significantly downregulated in the cancer tissues in comparison to normal tissue (FC = 0.022, *P* < 0.001) in GSE 104165 from GEO dataset. (**c**) The expression levels of miR-1246 were significantly higher in GBC patients with stage IIIB–IV in comparison to the Benign and HCs, but not in patients with stage 0–IIIA. Similarly, the miR-451a expression levels was significantly lower in GBC patients with stage III–IV, but not those with stage 0–II, in comparison to the Benign and the HCs. (**d**) The receiver-operating characteristic (ROC) curve analysis of miR-1246, miR-451a, CEA, and CA19-9 for discriminating GBC from Benign and HCs. These curves revealed that the optimal combination was CEA, CA19-9 and miR-1246 in serum EVs (sensitivity, 72.0%; specificity, 90.8%; accuracy, 81.7%; AUC, 0.816 [95% confidence interval, 0.712–0.888]). *P < 0.05 and **P < 0.01.

Following confirmation of strong positive-correlation between the results of microarray analysis and independent RT-qPCR (see Supplementary Fig. S1 online), miR-1246 and miR-451a were validated in all participants. Two remaining miRNAs, miR-29-1-5p and miR-31-3p, were excluded from further analyses due to Cq values of > 35. The miR-1246 expression in serum EVs was significantly higher in the GBC than in the Benign and the HCs (*P* = 0.005), while the miR-451a expression was significantly lower in the GBC in comparison to the Benign and the HCs (*P* = 0.001) (Fig. 3a). Similar results were also observed in the sub-analysis using serum samples collected within 5 years (see Supplementary Figs. S2, S3 online). Further investigation from the GEO database revealed that the miR-1246 and miR-451a expression in GBC tissue was significantly higher and lower, respectively in comparison to normal tissue (GSE104165: Fig. 3b. and GSE112408). In addition, the expression levels of the two miRNAs in the serum EVs were not significantly correlated with several blood test items, including the high density lipoprotein (HDL) level, or any type of hematocyte which may affect the expression levels of the miRNAs (see Supplementary Fig. S4 online)^19^, suggesting that serum EVs with upregulated miR-1246 and downregulated miR-451a in GBC might be derived from GBC tissue, and these distribution might be favorable for GBC progression, as demonstrated below.

### Potential application of miR-1246 and miR-451a in serum EVs as a diagnostic marker for GBC

By TNM stage (UICC), miR-1246 and miR451a were found to be associated with advanced-stage GBC (Fig. 3c).

Next, ROC curve analyses were performed to assess the potential application of these miRNAs in serum EVs as noninvasive biomarkers for diagnosis of GBC. The area under the curve (AUC) values of 0.646 (95% confidence interval [CI], 0.534–0.743) for miR-1246 and 0.664 (95% CI, 0.551–0.761) for miR-451a were revealed, while the AUC values of 0.770 (95%CI, 0.666–0.849) for CEA and 0.729 (95%CI, 0.613–0.822) for CA19-9 were revealed (Fig. 3d). Among these 4 biomarkers, the highest-precision combination was CEA, CA19-9 and miR-1246 (sensitivity, 72.0%; specificity, 90.8%; accuracy, 81.7%; AUC, 0.816 [95%CI, 0.712–0.888]) (see Supplementary Table 2 online).

### Potential application of miR-1246 in serum EVs as a prognostic marker in GBC patients

Overall survival (OS) was obviously better in stage 0–II patients than in stage III–IV patients among all GBC patients (median survival time: not reached, 401 days, and 175 days in stage 0–II, III and IV, respectively, *P* < 0.001) (see Supplementary Fig. S5 online). Thus, we focused on stage III–IV patients whose prognosis were relatively poor, and investigated the prognostic factors. A univariate analysis showed that an Eastern Cooperative Oncology Group performance status (ECOG PS) score: 1–3 (*P* = 0.004), presence of metastasis (*P* = 0.036), CEA ≥ 2 × ULN (upper limit of normal) (*P* = 0.047), CA19-9 ≥ 2 × ULN (*P* = 0.011) and higher expression level of serum EV miR-1246 (*P* = 0.003) were significantly associated with a poor prognosis in GBC patients. The multivariate analysis revealed that serum EV miR-1246 high and ECOG PS: 1–3 were significant independent prognostic factors (Hazard ratio [HR] 3.05 [95%CI: 1.22–7.81], *P* = 0.017 and HR 3.22 [95%CI: 1.18–8.54], *P* = 0.023, respectively) (Table 2).

**Table 2:** Prognostic factors for overall survival in gallbladder cancer patients with stage III–IV.

Variables	N (%)	Univariate analysis		Multivariate analysis	
		HR (95% CI)	P	HR (95% CI)	P
Age ≥ 73	21 (50)	1.01 (0.50–2.03)	0.975		
Male	24 (57)	1.20 (0.60–2.40)	0.598		
ECOG PS 1–3	12 (29)	2.91 (1.39–6.10)	0.004	3.22 (1.18–8.54)	0.023
UICC T4	15 (36)	1.64 (0.78–3.38)	0.176		
UICC N1-2	32 (76)	1.63 (0.77–3.81)	0.22		
UICC M1	16 (38)	2.15 (1.02–4.45)	0.036	0.92 (0.36–2.25)	0.864
Alb < 3.5	22 (52)	1.80 (0.89–3.69)	0.362		
AST ≥ 76 (2 × ULN)	12 (29)	1.76 (0.79–3.62)	0.146		
T-bil ≥ 5 (5 × ULN)	9 (21)	1.29 (0.54–2.79)	0.533		
NLR ≥ 3	22 (52)	1.31 (0.66–2.64)	0.434		
CEA ≥ 10 (2 × ULN)	14 (33)	2.09 (0.97–4.39)	0.047	1.20 (0.47–3.09)	0.708
CA19-9 ≥ 78 (2 × ULN)	21 (50)	2.56 (1.23–5.47)	0.011	1.56 (0.62–3.84)	0.334
Serum EV miR-1246 high	21 (50)	2.99 (1.41–6.45)	0.003	3.05 (1.22–7.81)	0.017
Serum EV miR-451 low	21 (50)	1.09 (0.55–2.21)	0.799		

ECOG, Eastern Cooperative Oncology Group; PS, Performance status; UICC, Union for International Cancer Control; Alb, albumin; AST, aspartate transaminase.

T-bil, total bilirubin; NLR, neutrophil-to-lymphocyte ratio; CEA, carcinoembryonic antigen; CA19-9, carbohydrate antigen 19-9; EV, extracellular vesicles.

ULN, upper limit of normal; HR, hazard ratio; CI, confidence interval.

### Upregulation of endogenous miR-1246 promotes cell proliferation and GBC cell invasion

**Figure 4:**
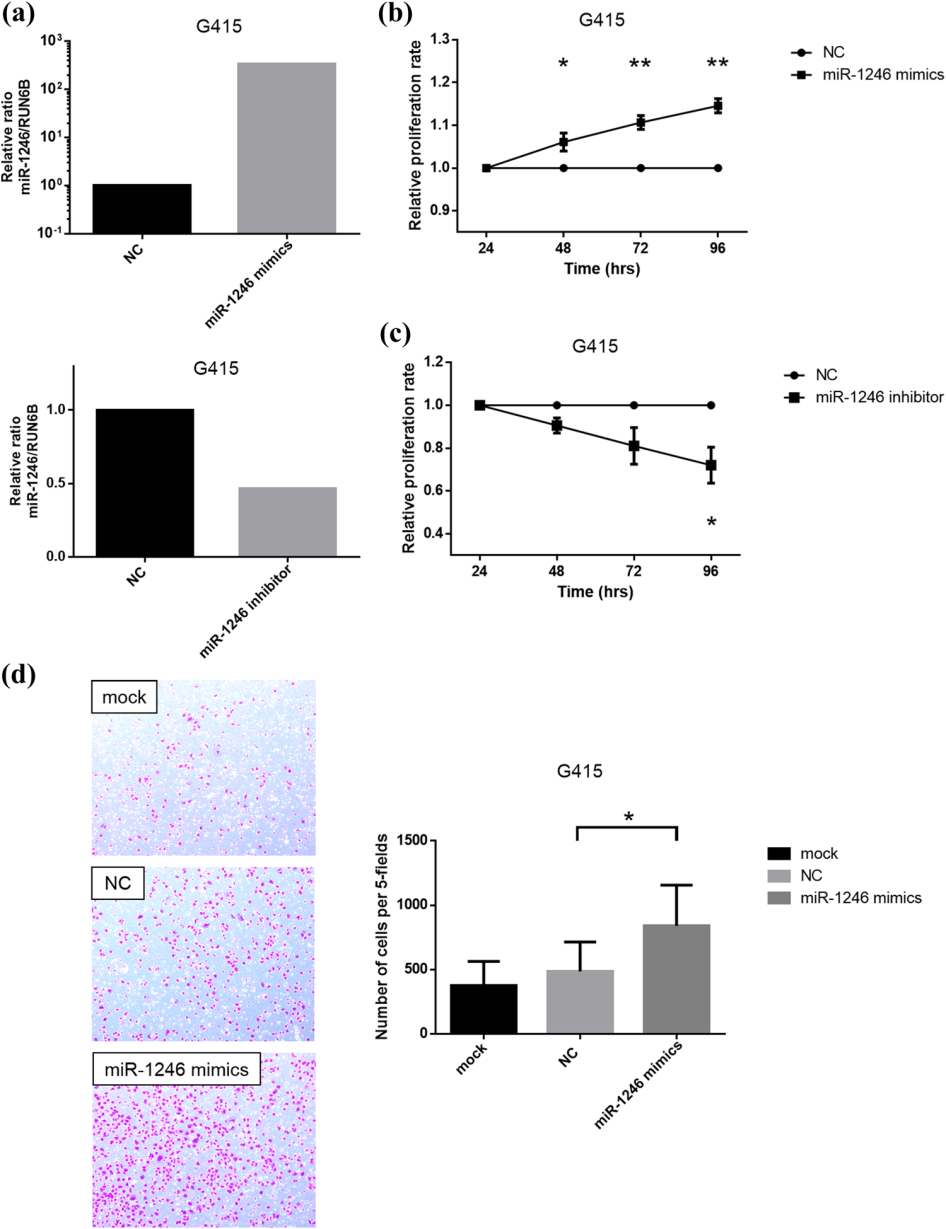
MiR-1246 promotes cell proliferation and invasion in gallbladder cancer cell lines. (**a**) Validation of the miR-1246 expression level after transfection of miR-1246 mimics, miR-1246 inhibitor and negative control in G415 cells was measured by RT-qPCR. The miRNA expression levels were normalized to the RNU6B expression. (**b**) The proliferation rate in G415 cells after transfection of 3 nM miR-1246 mimics or negative control was assessed by an MTT assay. The G415 cells showed significantly increased cell proliferation after the transfection of miR-1246 mimics. (**c**) The proliferation rate in G415 cells after the transfection of 100 nM miR-1246 inhibitor or negative control was assessed by an MTT assay. The G415 cells showed significantly decreased cell proliferation after the transfection of miR-1246 inhibitor. (**d**) Cell motility after the transfection of 3 nM miR-1246 mimics, negative control or mock was assessed by a Transwell invasion assay. The numbers of invaded cells significantly increased upon transfection of miR-1246 mimics in comparison to negative control. Data are presented as mean ± s.e.m. *P < 0.05 and **P < 0.01.

To clarify the biological significance of the above-described findings, the functions of miR-1246 in GBC cells were tested. As a result of miR-1246 mimics transfection (Fig. 4a), the proliferation rate of G415 cells transfected with miR-1246 mimics significantly increased (Fig. 4b), while that of G415 cells transfected with miR-1246 inhibitor significantly decreased relative to the controls (Fig. 4c). Furthermore, the number of invasive cells among the total G415 cells transfected with miR-1246 mimics was significantly higher than that in the controls (Fig. 4d). Taken together, the upregulation of miR-1246 promoted cell proliferation and invasion in G415 cells, suggesting a tumor-promoting role of miR-1246 in GBC cells.

### Upregulation of endogenous miR-451a inhibits cell proliferation and promotes GBC cell apoptosis

**Figure 5:**
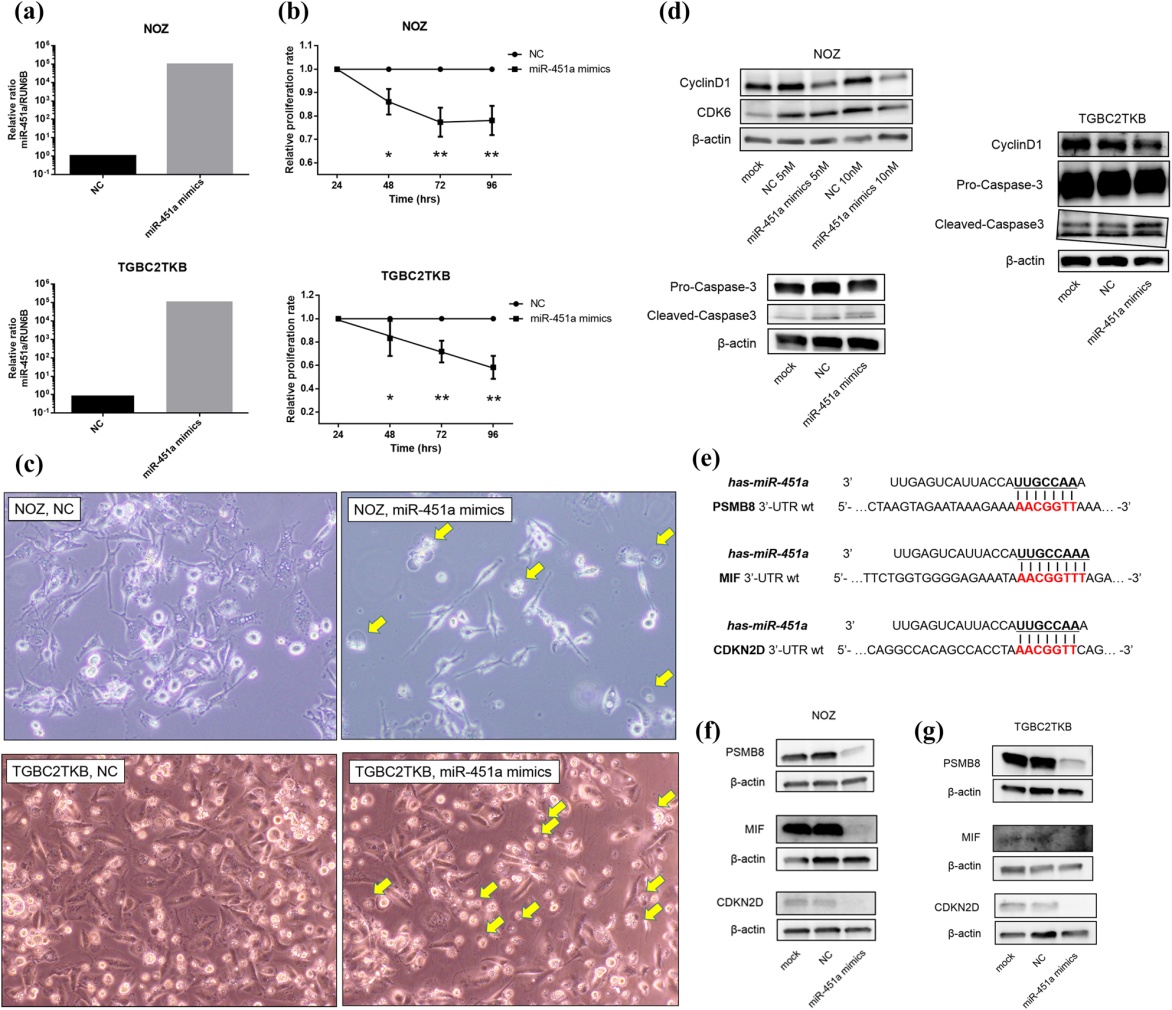
MiR-451a inhibits cell proliferation and induces apoptosis in gallbladder cancer cell lines. (**a**) Validation of the miR-451a expression level after the transfection of miR-451a mimics and negative control in NOZ cells and TGBC2TKB cells was measured by RT-qPCR. The miRNA expression levels were normalized to the RNU6B expression. (**b**) The proliferation rate in both NOZ cells and TGBC2TKB cells after the transfection of 10 nM miR-451a mimics or negative control was assessed by an MTT assay. Both cells showed significantly decreased cell proliferation after the transfection of miR-451a mimics. (**c**) Under a phase-contrast microscope, floating, round and chromatin-concentrated cells were observed after the transfection of 10 nM miR-451a mimics, suggesting that apoptosis could be induced by the miR-451a mimics in GBC cells. (**d**) Western blotting showed that cyclinD1 and CDK6 were reduced in both NOZ cells and TGBC2TKB cells after the transfection of miR-451a mimics, depending on the concentration of miR-451a mimics. In addition, increased cleaved-caspase-3 with decreased pro-caspase-3 was observed in both cells after transfection of miR-451a mimics. β-actin was used as a loading control. (**e**) MiR-451a binding sites in the 3’UTR of PSMB8 mRNA, MIF mRNA, and CDKN2D mRNA. (**f**) Using Western blotting, the protein expression levels of PSMB8, MIF and CDKN2D were confirmed to be reduced in NOZ cells after the transfection of 10 nM miR-451a mimics. β-actin was used as a loading control for Western blotting. (**g**) Western blotting showed that the protein expression levels of MIF, PSMB8 and CDKN2D were reduced in TGBC2TKB cells after the transfection of 10 nM miR-451a mimics. β-actin was used as a loading control. Full-length blots were presented in Supplementary Figs. 6, 9.

The functions of miR-451a in GBC cells were also tested. After transfection of miR-451a mimics into NOZ cells and TGBC2TKB cells (Fig. 5a), cell proliferation of both cells was significantly inhibited in cells transfected with the mimics in comparison to control (Fig. 5b). For further study of the effects of miR-451a on the cell cycle, the expression of cell cycle-related proteins was analyzed by Western blotting. The Cyclin D1 expression was notably decreased at 48 h in both cells transfected with miR-451a mimics, depending on the concentration of miR-451a mimics (Fig. 5d, see Supplementary Fig. S6 online). Catalytic subunits of cyclin D1, namely, Cdk6, were also decreased in the cells. Moreover, the emergence of DNA fragmentation was detected by a cell-cycle analysis (see Supplementary Fig. S7 online), and round and chromatin-concentrated cells were detected by phase-contrast microscopy (Fig. 5c), and upregulated cleaved caspase-3 with downregulated pro-caspase-3 were detected by Western blotting, suggesting that apoptosis was induced by the overexpression of miR-451a in these GBC cells. To identify genes targeted by miR-451a, we performed an in silico analysis using miRDB^20^, and 3 genes (macrophage migration inhibitory factor [MIF], proteasome subunit beta type-8 [PSMB8], and cyclin-dependent kinase 4 inhibitor D [CDKN2D]) with higher target prediction scores (90 for PSMB8, 82 for CDKN2D, and 78 for MIF) were reported to be directly associated with inhibition of cell proliferation and apoptosis among 40 predicted targets (Fig. 5e)^21–23^. Our data showed that the expression levels of both mRNAs and proteins of these three focused genes were significantly downregulated in GBC cells transfected with miR-451a mimics in comparison to control (Fig. 5f, g, see Supplementary Figs. S8, S9 online). This was also consistent with the TCGA sample data showing that the expression of these mRNAs was significantly higher than that in normal tissue (see Supplementary Fig. S10 online)^24^. Taken together, upregulation of miR-451a inhibited cell proliferation and induced apoptosis in NOZ and TGBC2TKB cells, partly through regulation of MIF, PSMB8 and CDKN2D, suggesting that miR-451a has a tumor-suppressing role and could be a novel therapeutic target in GBC cells.

## Discussion

We found that miR-1246 and miR-451a in serum EVs were significantly upregulated and downregulated in GBC patients, respectively, in comparison to benign GB patients and healthy controls, using a comprehensive miRNAs assay and RT-qPCR. Particularly, miR-1246 was found to be a promising diagnostic biomarker for GBC and an independent prognostic factor in GBC. In additional in vitro studies, the overexpression of miR-1246 promoted GBC cell proliferation and invasion and the inhibitor of miR-1246 suppressed the oncogenic feature. Interestingly, the overexpression of miR-451a inhibited cell proliferation and induced apoptosis with targeting of MIF, PSMB8 and CDKN2D, suggesting that miR-451a could be a novel therapeutic target in GBC. To our knowledge, this is the first report demonstrating the clinical application of miR-1246 in serum EVs as a diagnostic biomarker and an independent prognostic biomarker, and the potential of miR-451a replacement therapy for GBC.

EVs were actively secreted from cancer cells, and would play an important role in cell–cell interactions for cancer progression^25–27^. In addition, most detectable miRNAs in serum were concentrated in EVs^28^, and the EV concentration in cancer patients was higher than in healthy individuals^29^. Thus, while miRNAs in serum were sufficiently robust to serve as practicable clinical biomarkers^30^, the analysis of isolated EVs, which could include cancer-derived molecules, would provide more accurate, meaningful, and reproducible biomarkers, and would contribute to an early diagnosis. We found the significant upregulation of miR-1246 and downregulation of miR-451a in serum EVs in GBC patients in comparison to non-GBC individuals. These findings were predominant in GBC patients with advanced stage. Previous reports revealed that miR-1246 in circulating EVs was useful for the diagnosis of breast cancer^31^, non-small cell lung cancer^32^, colon cancer^27^, gastric cancer^33^, and prostate cancer^34^. Similarly, miR-451a in circulating EVs was reported to be a diagnostic marker in hepatocellular carcinoma^35^ and a non-invasive biomarker predicting recurrence and the prognosis in pancreatic cancer^36^ and lung cancer^37^. Further investigation is needed to know how much EVs isolated from serum is derived from cancer tissue, and to elucidate whether the aberrant expression of miRNA in circulating EVs is derived from cancer tissue itself with the tumor microenvironment or non-cancerous tissue, including an immune reaction^38^. Although absence of cancer-specific EVs marker makes the complete solution difficult, we clarified the positive correlations of the miR-1246 and miR-451a expression between serum EVs and tissue, indicating that the isolated serum EVs were partly secreted from cancer tissue, probably dependent on the stage of GBC.

Tumor markers, including CEA and CA19-9, have been used as diagnostic and prognostic markers for GBC patients and for gastric, colorectal, and pancreatic cancer patients^9^. However, these markers are affected by various clinical situations, including obstructive jaundice, cholangitis and cholecystitis; thus, their accuracy is often low. In this study, there was no correlation between miR-1246 in serum EVs and serum hepatobiliary enzymes, and the combination of CEA and CA19-9 with miR-1246 improved the accuracy (sensitivity, > 10%; specificity, no change) for the diagnosis of GBC in comparison to each single marker. Furthermore, the elevated miR-1246 expression in serum EVs was identified as a novel prognostic factor for a poor PS. These results demonstrated that miR-1246 in serum EVs was a useful assistive-marker for discriminating GBC from non-cancerous lesion, which would also reflect the state of cancer itself.

Next, we clarified the functional role of miR-1246 and miR-451a, which could be conferred from cell to cell via EVs, for the progression of GBC. We found that miR-1246 regulated GBC cells as an oncogenic factor. MiR-1246 has been identified as a tumor promoter in several types of cancer^39–41^. In cervical cancer, miR-1246 promoted cell proliferation, migration, and invasion through suppression of its target gene thrombospondin 2^39^, and the expression in serum in patients with lymph node metastasis was significantly upregulated in comparison to those without metastasis^40^. In lung cancer, miR-1246 promoted migration, invasion, and EMT by regulating the Wnt/β-catenin pathway through directly targeting GSK-3β/β-catenin, which partly contributed to metastasis^41^. Additionally, the miR-1246 expression was associated with chemoresistance and cancer stem cell-like properties via cyclin-G2, and predicted a worse prognosis in pancreatic cancer patients^42^. Interestingly, Sakha et al. reported that miR-1246 packaged into EVs was delivered between metastatic oral squamous cancer cell lines and induced increased cell motility and invasion directly through the regulation of DENN domain-containing protein 2D^18^. These findings support that GBC tissues secrete cancer-specific EVs containing abundant miR-1246, and EVs transfer between cancer cells, inducing progression and metastasis. However, further research is needed to discover specific target genes in GBC and to elucidate whether such mechanisms using cell-to-cell communication via the EVs are truly activated. In addition, miR-451a, which was downregulated in cancer tissues, has been shown to act as tumor suppressor in many types of cancer. These findings are consistent with our results demonstrating that the overexpression of miR-451a inhibited cell proliferation and apoptosis in GBC cell lines. For instance, the upregulation of miR-451a inhibited cell proliferation in papillary thyroid cancer^17^, renal cell carcinoma^43^, and lung cancer^44^, induced apoptosis^17,43^ and suppressed EMT^17^ and metastasis^44^, by targeting PSMB8. MiR-451a can inhibit cell proliferation, migration, and angiogenesis, and promotes apoptosis of osteosarcoma^21^, and suppresses cell proliferation and migration in non-small cell lung cancer cells, by inhibiting MIF and the expression of phosphorylated Akt^22^. In addition, miR-451a inhibits the proliferation of EC9706 cells by targeting CDKN2D and MAP3K1^23^. According to the miRDB database, these 3 proteins were strongly predicted to be targets of miR-451a, although another unknown target that can be regulated toward inhibition of tumorigenicity may exist. Our data also showed an inverse relationship between miR-451 and the expression of these proteins. In addition, the re-expression of miR-451a could serve to release cells from chemoresistance and radio-resistance^45^. Thus, miR-451 could be an extremely promising target for miR-mimics based therapy, as a miR-16-based mimic drug reportedly exerted antitumor activity in malignant pleural mesothelioma patients^46^. We will investigate the efficacy of miR-451a mimics treatment in vivo, by the local injection of miR-mimics complexed with non-toxic atelocollagen^47^ which may be more suitable to target the tumor cells, without unpredictable adverse effects induced by intravenous injection, and using an organoid model to clarify the effectiveness against cancer stem cells.

In the interpretation of our study, we note a few important considerations regarding the method of serum EV isolation and sample selection for achieving more precise results. First, mini-SEC was used for the isolation of EVs. SEC has multiple advantages over ultracentrifugation, including high particle yields, high-purity, reproducibility, cost-effectiveness, and nondestructive outcomes^14,48^. SEC is also comparable to density gradient purification of EVs^49^. Furthermore, contamination of miRNAs complexed with HDL was mostly excluded based on the size-based isolation method. Second, the miR-451a levels in red blood cells were significant, and were proportional to the degree of hemolysis^50^. Accordingly, we excluded hemolytic samples using oxyhemoglobin absorbance, and confirmed that the absence of correlation between miR-451a and peripheral blood hematocytes was observed as shown in Supplementary Fig. S4.

The present study was associated with some limitations. First, serum samples had stored for many years might cause some biases. However, a similar result was obtained using serum samples collected within 5 years, and a report established by Ge et al. supported our result; the report, using pooled sample from the same patient revealed that miRNAs including miR-451a in plasma EVs was stable, even though the plasma was stored for 5 years^51^. Thus, further studies are needed to verify the biomarkers in a multi-center prospective study, in a larger cohort, especially GBC patients with early-stage disease. Second, although the expression of miR-451a in serum EVs in GBC patients increased in microarray assay, its expression in RT-qPCR was significantly lower. As shown in Supplementary Fig. S1, the strong positive-correlation between both of them was confirmed (R^2^ = 0.9453), therefore, this conflicting results would arise due to small sample size in this exploratory analysis. Third, the miR-1246 and miR-451a expression in serum EV was not compared to that in tissue in individual cases, but the miRNAs levels were in accordance with the tissue levels in the GEO dataset. Fourth, miR-1246 and miR-451a are not specific GBC markers, as they have been identified as diagnostic and prognostic biomarkers in other types of cancers. However, they can be powerful tool for the diagnosis of GBC, in combination with traditional tumor markers, advanced imaging findings of GB wall-thickening and polypoid lesions, and concomitant congenital pancreatobiliary-maljunction, which is a risk factor of GBC. Fifth, the methods of EV isolation are not standardized and there is no established method for absolute quantification of miRNA in biofluid; thus, we adapted a spike-in normalization approach to control for technical variance and data normalization, based on previous reports^52^. This method would serve relative amounts of each miRNA in a certain quantity of sample faithfully, without the influences of other miRNAs, including miR-16, which is often used as an internal control for serum samples, but might be degenerated by RNase.

In conclusion, we clarified the clinical relevance and potential roles of miR-1246 and miR-451a in GBC, by analyzing serum EV samples, tissues and GBC cell lines transfected with the miRNAs. We revealed that miR-1246 and miR-451a in serum EVs possibly derived from GBC cancer tissue showed aberrant expression levels in GBC patients in comparison to non-GBC individuals, thus indicating these miRNAs are potentially useful as diagnostic and prognostic biomarkers in the clinical setting. Furthermore, these miRNAs has potential roles for GBC progression, and miR-451a was proposed as a promising therapeutic target for GBC. Further investigation for the diagnosis of early GBC and the development of miR-mimics based therapy is strongly desired.

## Methods

### Serum samples

Peripheral blood samples were obtained from 55 patients with GBC, 50 with Benign, and 14 HCs at Okayama University Hospital and Okayama Saiseikai General Hospital in 2007–2019. All patients were pathologically diagnosed with malignant or benign disease based on surgery or EUS-guided biopsy. Patients with malignant disease in the past five years, neuroendocrine tumor of the gallbladder, hemolyzed serum samples (judged by oxyhemoglobin absorbance at 414 nm)^50^, or whose serum samples were lacking, were excluded. Blood samples were collected before any treatments, including surgery, chemotherapy and radiotherapy. Collected blood was centrifuged for 15 min at 3000 rpm, and the serum was stored below – 30 ℃. This study was approved by the Okayama University Human Ethics Committee (Approval number: 1908-052) and informed consent was obtained from all participants. All experiments were performed in accordance with the relevant national guidelines and regulations.

### Serum EVs isolation using size-exclusion chromatography

Serum (500 µL) was centrifuged at 2000×*g* for 10 min, and then 10,000×*g* for 30 min at 4℃. Clarified serum was further filtered using a 0.22-µm pore filter to remove large microvesicles and large lipoproteins, added to phosphate-buffered saline (PBS) to make a total 1 ml of preparation, and used for subsequent EV isolation^2^. SEC-based isolation, called “mini-SEC” because it utilized small disposable columns, was conducted according to previous reports^14,48^. In brief, Sepharose 2B (Sigma-Aldrich, St. Louis, MO, USA) was packed into 1.5 cm × 12 cm mini-columns (Bio-Rad, Hercules, CA, USA; Econo-Pac columns), with a column bed volume of 10 ml. After washing the column with PBS, 1 ml of clarified serum was loaded onto the column and the eluate was considered as fraction #0. Subsequently, 1 ml of PBS was repeatedly added, and fraction #4 was collected for a downstream analysis because this major fraction contained unclustered morphologically-intact EVs.

### Characterization of isolated serum EVs

The morphology of EVs was observed by transmission electron microscopy (TEM) after preparation. Briefly, 10 μl of EVs samples were applied on Formvar-carbon coated TEM grids and left aside to allow membranes adsorb for 15 min. The grids were stained with 2% uranyl acetate for 2 min. Finally, TEM (H7650, Hitachi, Japan) was used for imaging at Central Research Laboratory, Okayama University Medical School. Size distribution of EVs was measured using a Zetasizer Nano ZS system (Malvern Instruments, Malvern, U.K.) at Okayama Medical Innovation Center, and the data were analyzed using Zetasizer Software (V7.03) (Malvern Instruments). Protein concentrations were determined using a BCA protein assay (Pierce Biotechnology, Rockford, IL, USA) according to the manufacturer's instructions. In all cases, fraction #4 was applied for these characterizations.

### miRNA microarray analysis

Total RNA was extracted from 300 µL of EV sample using the 3D-Gene RNA Extraction Reagent (Toray Industries, Tokyo, Japan) according to the manufacturer's instructions, and was checked the quality using the Agilent RNA 6000 Pico Kit and Agilent 2100 Bioanalyzer (Agilent Technologies, Palo Alto, CA, USA). A comprehensive miRNA expression analysis was performed using a 3D-Gene miRNA Labeling kit and a 3D-Gene Human miRNA Oligo Chip (Toray Industries), which was designed to detect 2588 miRNAs registered in miRBase release 21. Fluorescent signals were scanned with the 3D-Gene Scanner 3000 and analyzed using the 3D-Gene Extraction software program (Toray Industries). The global normalization method for the background-subtracted signal intensities was used so that the median of these signal intensities became 25.0. We calculated the fold-change (FC) values of the GBC for each miRNA using the signals of Benign and HCs as a reference.

The microarray data were obtained from 3 GBC samples, 3 Benign samples and one pooled sample of 10 HCs for an exploratory analysis. We selected candidate miRNAs with the following conditions: FC > 2 and known as a potential miRNA associated with cancer progression or EMT in previous articles.

### Quantitative reverse transcription polymerase chain reaction (RT-qPCR)

Total RNA was extracted from 200 μl of EV samples using a miRNeasy Micro Kit (Qiagen, Valencia, CA, USA) for RT-qPCR, according to the manufacturers’ instructions. To improve the RNA yield, 1 μg of RNA carrier (MS2 bacteriophage RNA [Roche Applied Science]) was applied, and 2 fmol of a synthetic C. elegans miRNA cel-miR-39 (1 μl of 2 nM) (Qiagen) was added to denatured samples for normalization of sample-to-sample variation. Total RNA was eventually eluted by adding 60 μl of RNase-free water. Next, total RNA samples were reverse transcribed using the TaqMan MicroRNA Reverse Transcription Kit (Applied Biosystems, Foster City, CA, USA) and each miRNAs primer. Other primers for gene expression assay can be also found in the Supplementary Methods. RT-qPCR was performed using TaqMan Fast Advanced Master Mix and a LightCycler 96 Real-Time PCR System (Roche, Basel, Switzerland) in 96-well plates. Amplification curves were analyzed using the Roche LC software program, and were used to establish PCR amplification efficacy. All reactions were performed in duplicate.

### Cell culture and transfection of miRNAs

The human GBC cell lines, G415, NOZ and TGBC2TKB were obtained from Tohoku University (Sendai, Japan), JCRB cell bank (Osaka, Japan) and RIKEN cell bank (Tsukuba, Japan), respectively. NOZ was established by Dr. S. Nagamori (National Institute of Infectious Diseases, Japan). These were cultured in RPMI-1640 (Gibco) supplemented with 10% fetal bovine serum (FBS), William’s E Medium (Gibco) supplemented with 10% FBS and L-glutamine, and Dulbecco's Modified Eagle Medium (Gibco) containing low glucose and supplemented with 5% FBS, in a humidified atmosphere containing 5% CO_2_ at 37℃.

For gain-of-function or loss-of-function by miRNA transfection, cells were seeded at 12–16 × 10^4^ cells/well in a 24-well plate, pre-cultured in the medium containing 10% FBS until 60% confluency and then transfected with mirVana™ miRNA mimics (has-miR-451a [MC10286], has-miR-1246 [MC13182] and negative control #1 [4464058]) (Thermo Fisher Science) or mirVana™ miRNA inhibitor (has-miR-1246 [MH13182] and negative control #1 [4464078]) using Lipofectamine™ RNAiMAX Transfection Reagent (Invitrogen, Carlsbad, CA, USA) following the manufacturer's protocol. Cells were cultured for 48 h, and then used for downstream assay.

### MTT assay

Cellular proliferation was measured by an MTT (3-[4,5 dimethylthiazol-2-yl]-2, 5-diphenyltetrazolium bromide) assay. Briefly, cells were seeded in 96-well plates at a density of 2500 or 5000 cells/well, depending on cell lines, after 48 h of transfection. After 24, 48, 72 or 96 h of incubation, MTT (0.5 mg/ml in media) was added to each well, the cells were incubated for 3 h at 37℃, and the purple-blue formazan precipitate was dissolved using 200 μl of DMSO. Absorbance was read at 570 nm using a microplate reader, MULTISKAN GO (Thermo Fisher Scientific, Inc., Waltham, MA, USA). Media containing MTT reagent but without cells was used as a blank control. All experiments were performed in triplicate.

### Invasion assay

Transwell invasion assays were conducted by a two-chamber assay using an 8-μm pore, 24-well FALCON Cell Culture Insert pre-coated with Matrigel (Corning). Cells were transfected with hsa-miR-1246 mimics or negative control, and were cultured for 48 h. Then, 40,000 cells per chamber were plated, and cellular invasion was assessed at 16 h after plating. Cells invading the lower surface of filters were fixed, stained using 0.5% crystal violet, and counted in 5 random high-power fields. All experiments were performed in triplicate.

### Western blotting

Ten micrograms of harvested cell protein was resolved by SDS-PAGE and transferred to PVDF membranes (Bio-Rad, Hercules, CA, USA) using semi-dry transfer. The membranes were blocked using PVDF blocking reagent Can Get Signal™ (Toyobo, Osaka, Japan) for 1 h. Reactive bands were detected using Clarity Western ECL Substrate (#1705060: Bio-Rad) and the ImageQuant software program. The antibodies used in this study are listed in the Supplementary Methods.

### Bioinformatics analysis

To reveal miRNA expression in tissue, GEO datasets with the keywords “gallbladder carcinoma” and “miRNA” were used; then, GSE104165 and GSE112408 were enrolled. The miRNAs of interest were calculated to reveal their expression levels in GBC and normal tissue using GEO2R, an online analysis tool in the GEO database. The targets of miRNAs were identified by an online database, miRDB^20^. The expression profiles of target genes in CCC with data from The Cancer Genome Atlas were analyzed with UALCAN^24^.

### Statistical analysis

All statistical analyses were performed using JMP 15.0 (SAS Institute Inc, North Carolina, USA) or GraphPad Prism 6.0 (GraphPad Software, USA). Comparisons between groups were analyzed by the Kruskal–Wallis test, Mann–Whitney U test, Pearson’s chi-squared test or Wilcoxon rank sum test. The receiver-operating characteristic (ROC) was used to evaluate the utility of candidate miRNAs as diagnostic biomarkers of GBC. The cutoff values of miR-1246 and miR-451a were based on the median values. The survival rate was calculated using the Kaplan–Meier method with a log-rank (Mantel-Cox) test. Cox’s proportional hazard regression model was used to evaluate the prognostic factors. All tests were two-sided. *P *values of < 0.05 were considered statistically significant.

### Ethics approval and consent to participate

Serum samples were collected at Okayama University Hospital and Okayama Saiseikai General Hospital, and informed consent was obtained from all participants for this study. This study was approved by the Okayama University Human Ethics Committee (Approval number: 1908-052). The procedures involved human participants were done in accordance with the principles of the 1964 Declaration of Helsinki and its later amendments or comparable ethical standards.

### Consent to publish

This manuscript does not contain any individual patient data.

## Supplementary Information

Supplementary Information.

## Data Availability

The datasets generated and analyzed during the current study are available from the corresponding author on reasonable request.
