# Supplementary material for: Extracellular vesicle-shuttled miRNAs as a diagnostic and prognostic biomarker and their potential roles in gallbladder cancer patients
Source: Sci Rep. 2021 Jun 10;11:12298. doi: 10.1038/s41598-021-91804-0 (PMC8192895; doi:10.1038/s41598-021-91804-0)
Supplement: Supplementary file 1 — Supplementary Information. [file 41598_2021_91804_MOESM1_ESM.pdf]

Supplementary Information

Extracellular vesicle-shuttled miRNAs as a diagnostic and prognostic biomarker and their potential roles in gallbladder cancer patients

Eijiro Ueta, M.D.,[1] *Koichiro Tsutsumi, M.D, Ph.D.,[1] Hironari Kato, M.D., Ph.D.,[1]

Hiroshi Matsushita, M.D., Ph.D.,[1] Hidenori Shiraha, M.D., Ph.D.,[1]

Masakuni Fujii, M.D., Ph.D.,[2] Kazuyuki Matsumoto, M.D., Ph.D.,[1]

Shigeru Horiguchi, M.D., Ph.D.,[1] Hiroyuki Okada, M.D., Ph.D.[1]

[1]Department of Gastroenterology and Hepatology, Okayama University Hospital, Okayama, Japan;

[2]Department of Internal Medicine, Okayama Saiseikai General Hospital, Okayama, Japan

Correspondence: Koichiro Tsutsumi, M.D., Ph.D.

E-mail: tsutsumi@okayama-u.ac.jp

Supplementary Methods

Cell cycle analysis

Cells were transfected with hsa-miR-451a mimics or negative control, and cultured for 48 h. Samples were collected and fixed in 70% ethanol overnight. For a cell cycle analysis, fixed cells were washed in PBS and treated with RNase for 20 minutes at 37°C. Propidium iodide (PI; 5 μg/mL) was added to each sample and analyzed by fluorescence-activated cell sorting (FACS).

Antibodies

The primary antibodies used in this study were as follows: Cyclin D1 (#2922: Cell Signaling Technology), CDK6 (#3136; CST), Caspase-3 (8G10) (#9665: CST), Cleaved Caspase-3 (Asp175) (#9661: CST), MIF (sc-271631: Santa Cruz Biotechnology), PSMB8 (sc-365699: SCB), CDKN2D (sc-1665: SCB) and β-actin (#4967: CST). Horseradish peroxidase (HRP)-conjugated anti-mouse antibody (#7076: CST) or HRP-conjugated anti-rabbit antibody (#7074; CST) were used as secondary antibodies.

Primers for RT-qPCR

Taqman miRNA assay kit, such as has-miR-29b-1-5p [002165], has-miR-31-3p [002113], has-miR-451a [001141], has-miR-1246 [custom order], cel-miR-39 [000200]) and RNU6B

(001093) (Applied Biosystems, Carlsbad, CA, USA) was used. The forward and reverse primers used for each human gene were as follows: MIF: 5- ACAGCATCGGCAAGATCGG-3' and 5'- TAATAGTTGATGTAGACCCTGTCCG-3', PSMB8: 5'-TTACCTGCTTGGCACCATGT-3' and 5'- GCAGATAGTACAGCCTGCATTC-3', β-actin: 5'-CAGCTTCTTTGCAGCTCCTT-3' and 5'-GCAGCGATATCGTCATCCAT-3'.

Supplementary Figure S1

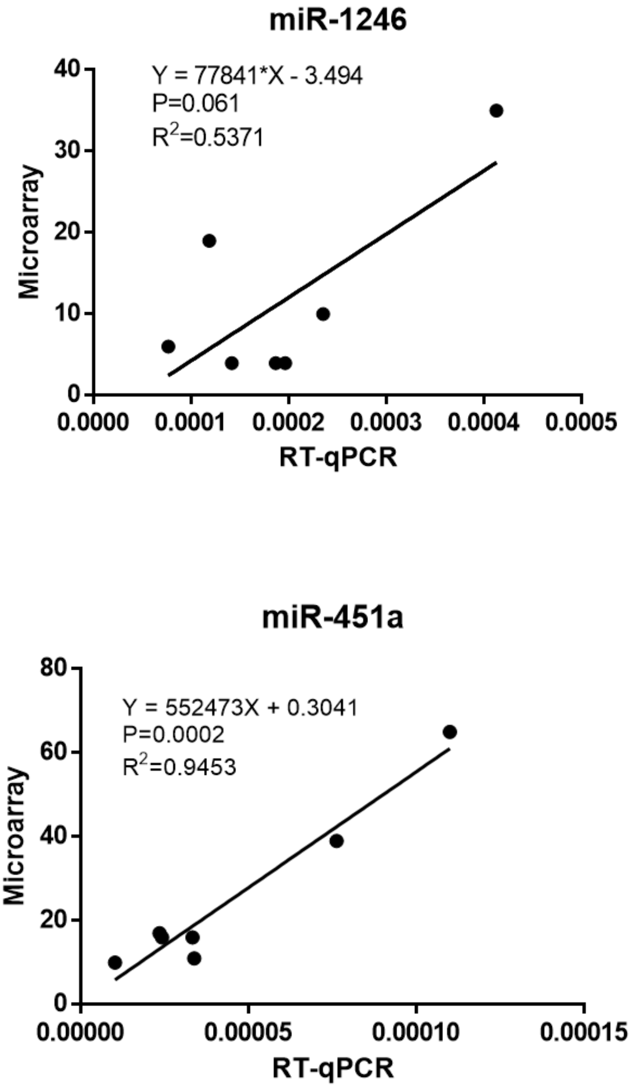

Supplementary Figure S1 A positive correlation in the expression levels of miR-1246 and miR-451a in serum EVs was observed between the comprehensive analysis and RT-qPCR.

Supplementary Figure S2

(a)

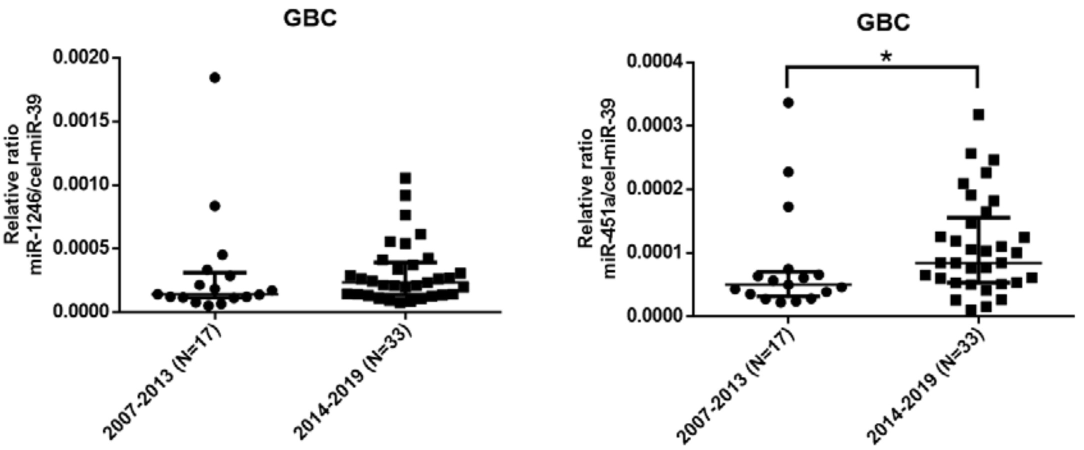

(b)

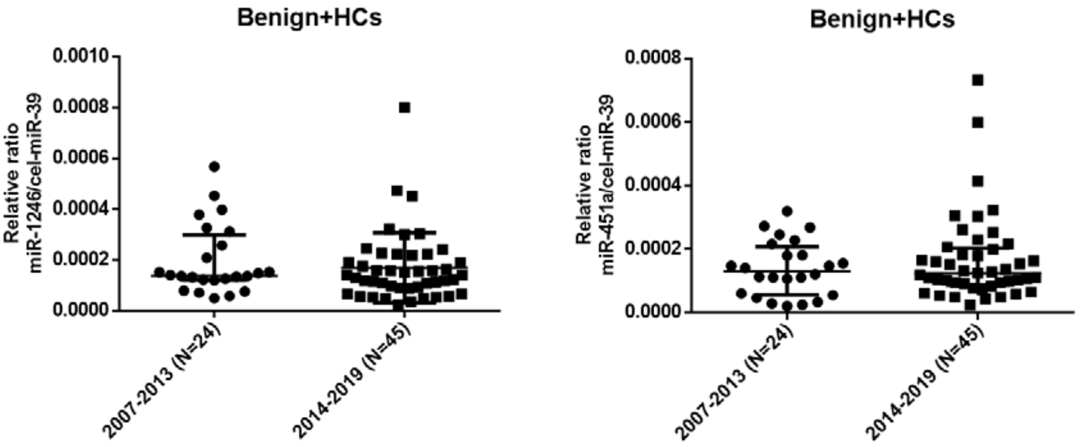

Supplementary Figure S2 The miR-1246 and miR-451a expression levels in serum EVs according to the period of serum collection

(a) In the GBC group, the miR-1246 expression levels in serum EVs did not differ between the early period (2007-2013) and the late period (2014-2019), while the miR-451a expression levels in the early period were significantly lower than those in in the late period (P=0.152 and P=0.035). (b) The expression levels of miR-1246 and miR-451a in serum EVs in the Benign and HCs group did not differ to a statistically significant extent between the periods (P=0.355 and P=0.776). GBC, gallbladder cancer; Benign, benign gallbladder disease; HCs, healthy controls.

Supplementary Figure S3

(a)

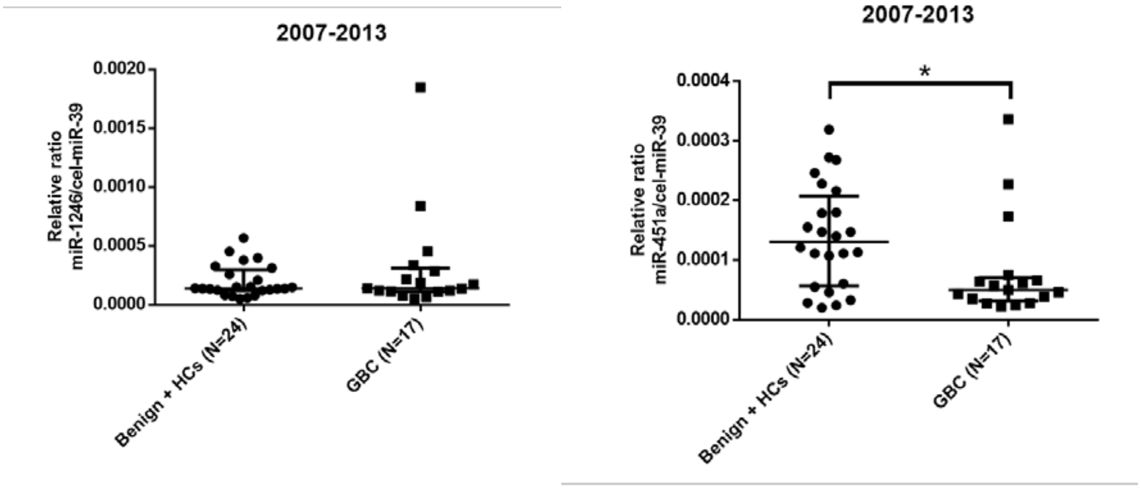

(b)

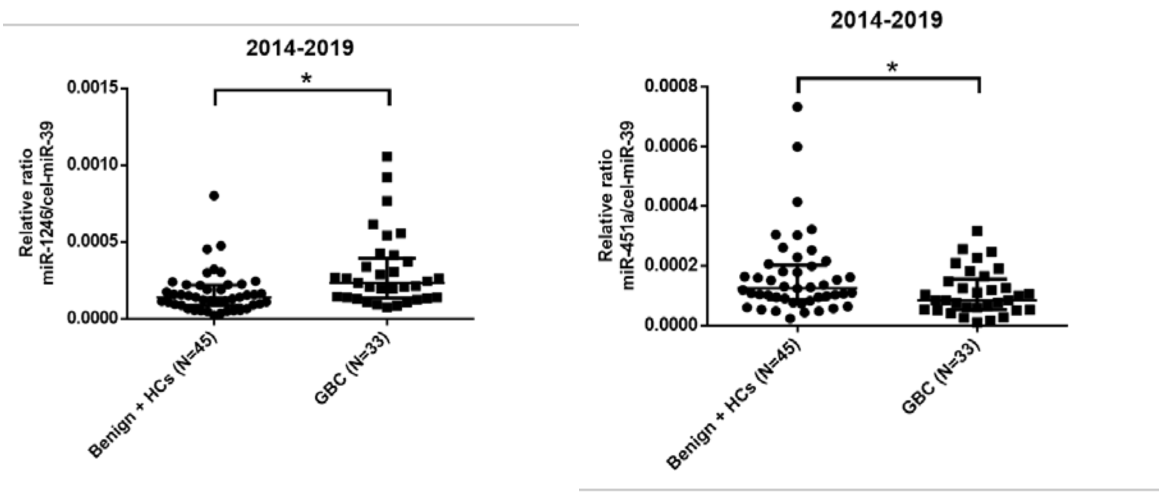

Supplementary Figure S3 The comparison of the miR-1246 and miR-451a expression levels in serum EVs between the GBC group and the Benign and HCs group according to period of serum collection

(a) In the early period, the miR-451a expression levels in the GBC group were significantly lower than in the Benign and HCs group (P=0.026); however, the miR-1246 levels in the two groups did not differ to a statistically significant extent. (b) In the late period, the miR-1246 expression levels in the GBC group were significantly higher than in the Benign and HCs group (P<0.001), while the miR-451a expression in the GBC group was significantly lower in comparison to the Benign and HCs group (P=0.040). This result was similar to the results of the original analysis, which was conducted using all samples in the whole period, as shown in Fig. 3a.

Supplementary Figure S4

(a)

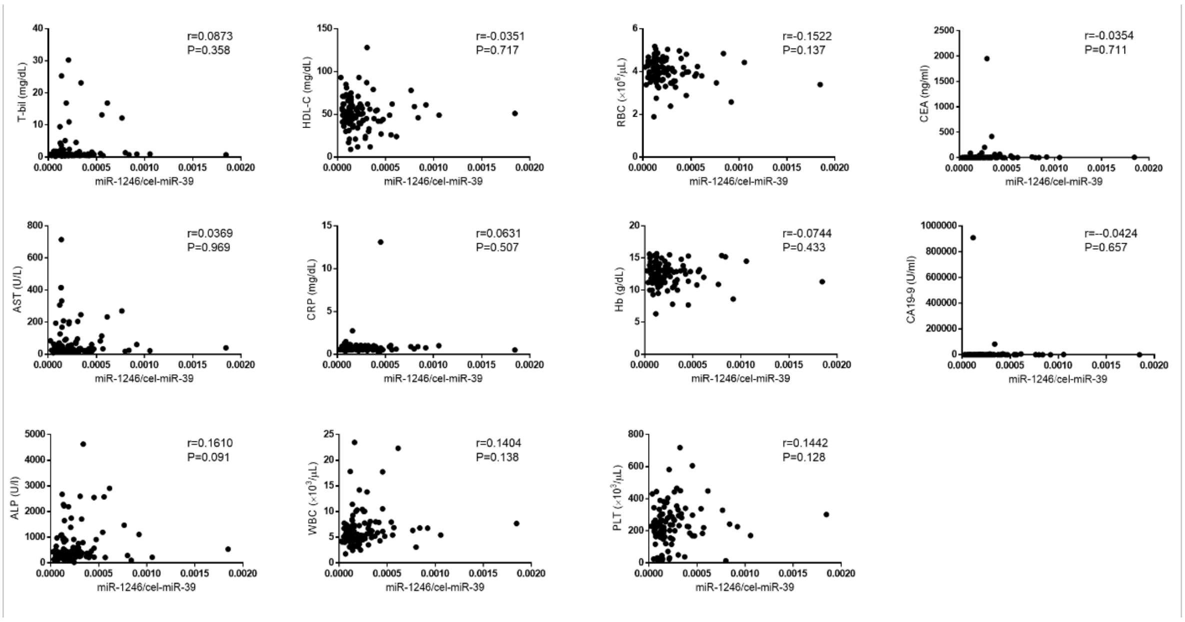

(b)

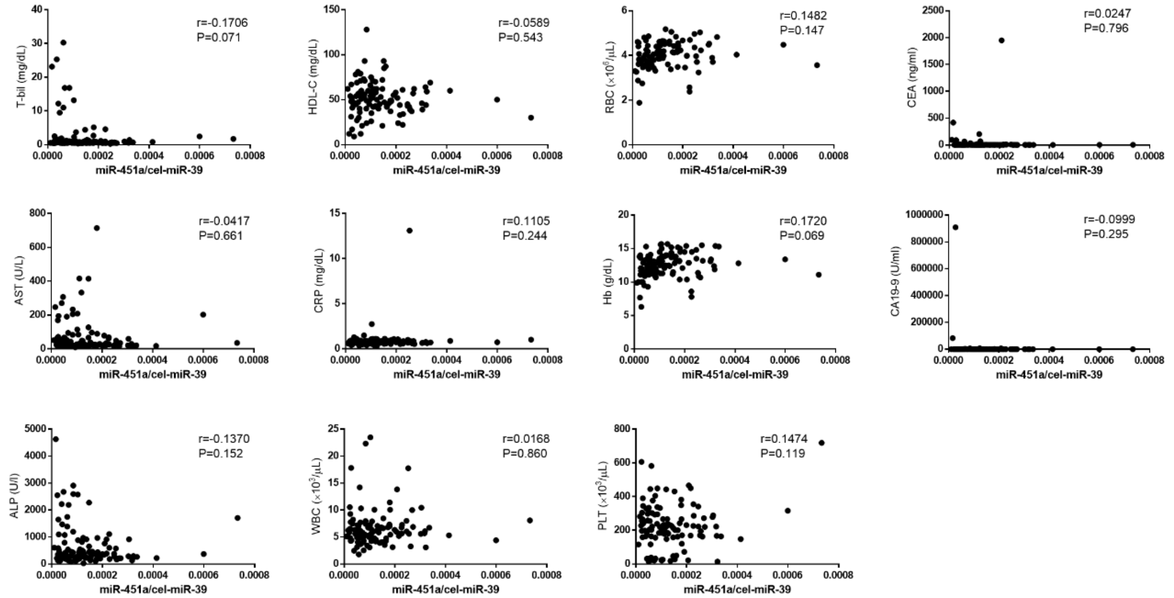

Supplementary Figure S4 Correlation between the expression levels of miRNAs in serum EVs and the peripheral blood biochemistry values in all participants. (a) There was no significant correlation between the levels of miR-1246 in serum EVs and the levels of total bilirubin (T-bil), aspartate aminotransferase (AST), alkaline phosphatase (ALP), high density lipoprotein cholesterol (HDL-C), C-reactive protein (CRP), white blood cell (WBC) count, red blood cell (RBC) count, hemoglobin (Hb), or platelet (PLT) count, as shown in the Pearson's correlation scatter plot. (b) Similarly, there was no significant correlation between the miR-451a levels in serum EVs and the levels of these examination items.

Supplementary Figure S5

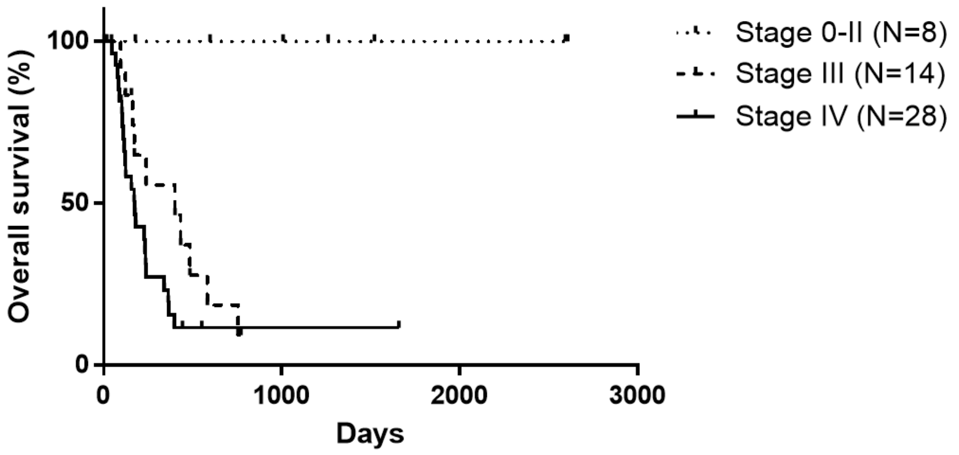

Supplementary Figure S5 Overall survival (OS) in patients with gallbladder cancer, as evaluated by Kaplan-Meier curves. OS in gallbladder cancer patients with stage Ⅲ-Ⅳ was obviously worse in comparison to patients with stage 0-Ⅱ (median survival time: in stage 0-Ⅱ, not reached; stage Ⅲ, 401 days; stage Ⅳ, 175 days; P<0.001, Mantel-Cox test).

Supplementary Figure S6

Fig. 5d

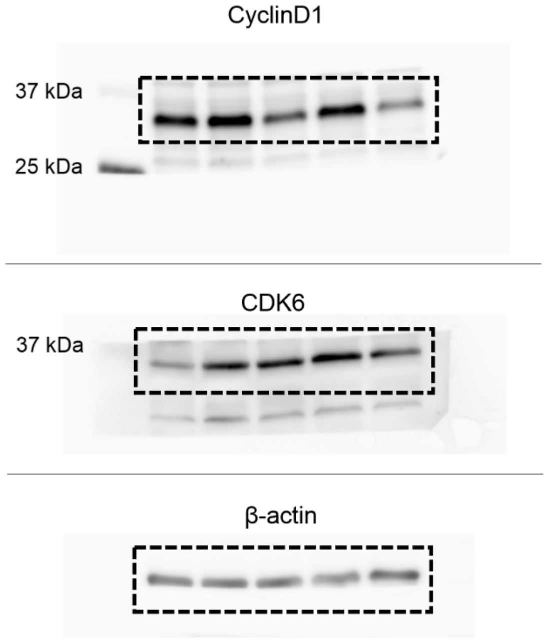

Fig. 5d

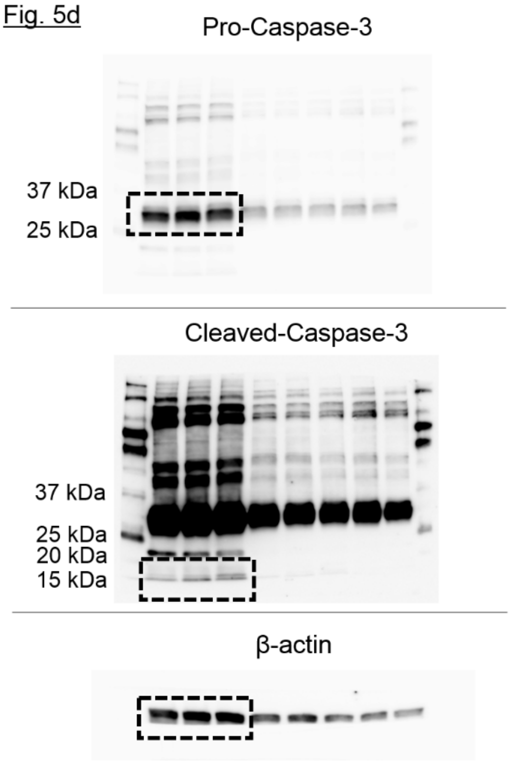

Fig. 5d

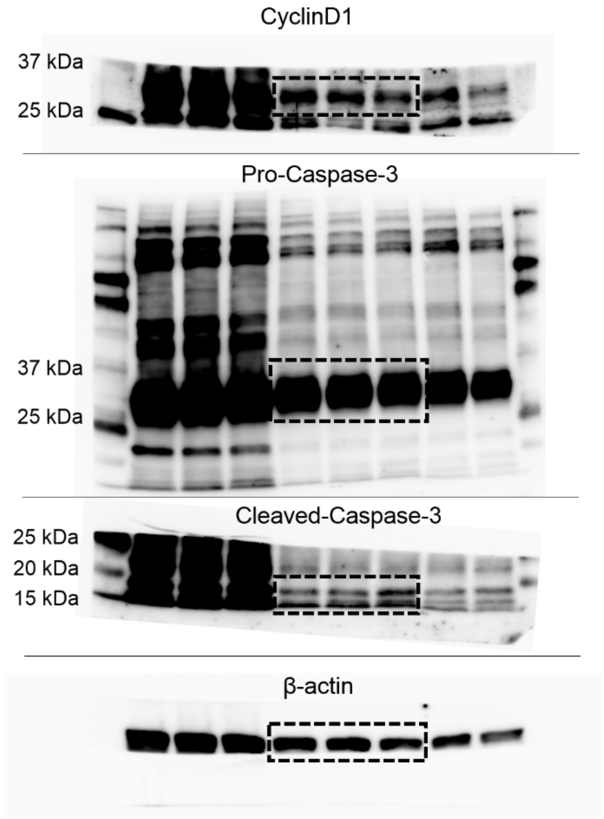

Supplementary Figure S6 Full blot images of Figure 5d

Supplementary Figure S7

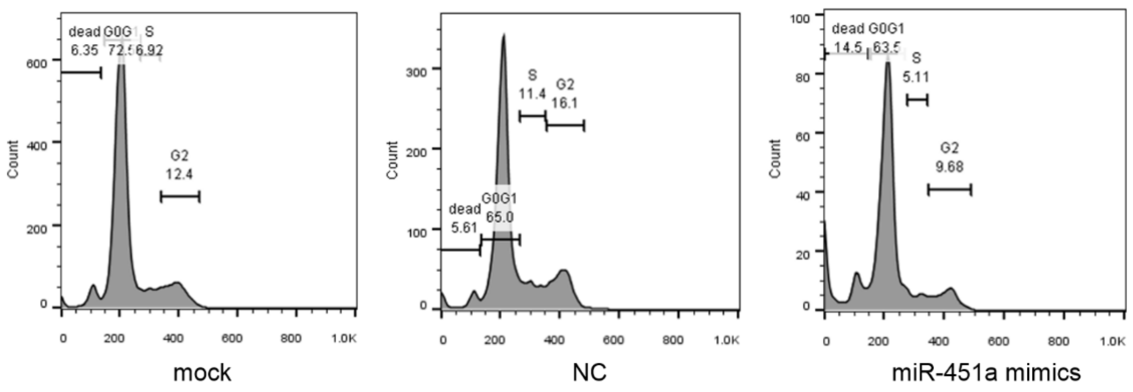

Supplementary Figure S7 The cell cycle analysis of NOZ cells after the transfection of miR-451a mimics. The cell cycle distribution of NOZ cells at 48 h after the transfection of 10 nM miR-451a mimics, negative control (NC) or mock revealed that no cell arrest under any of the conditions. However, the emergence of DNA fragmentation was confirmed, suggesting that apoptosis in NOZ cells could be induced by the miR-451a mimics.

Supplementary Figure S8

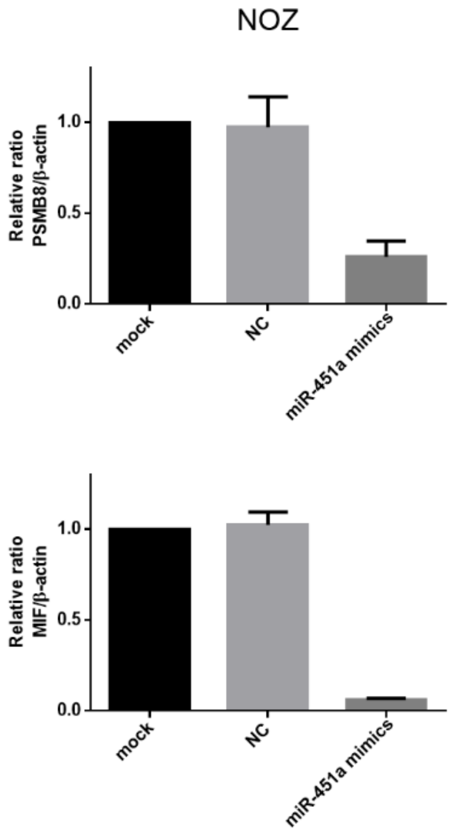

Supplementary Figure S8 The mRNA expression levels of PSMB8 and MIF in NOZ cells after the transfection of 10 nM miR-451a mimics, negative control (NC) or mock. β-actin was used as an internal control for RT-qPCR.

Supplementary Figure S9

(a)

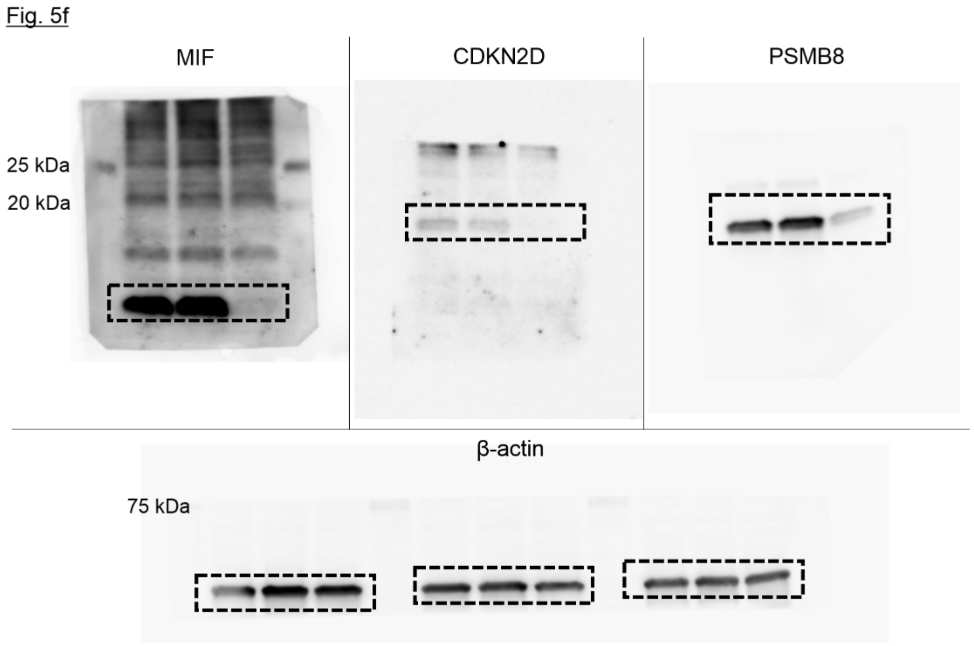

(b)

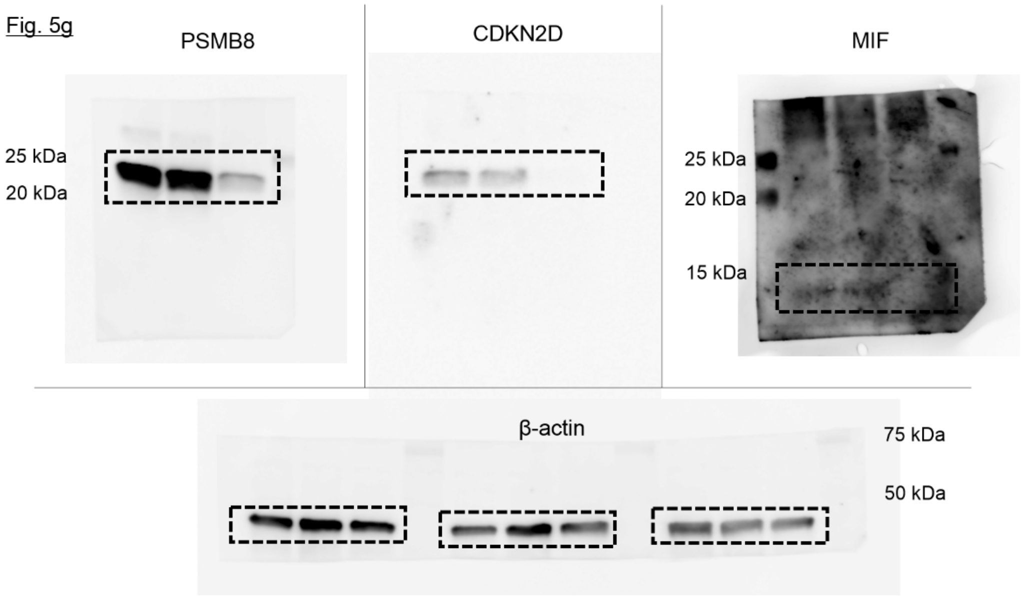

Supplementary Figure S9 Full blot images of Figure 5f and Figure 5g
(a) Uncropped images of Fig. 5f, (b) Uncropped images of Fig. 5g

Supplementary Figure S10

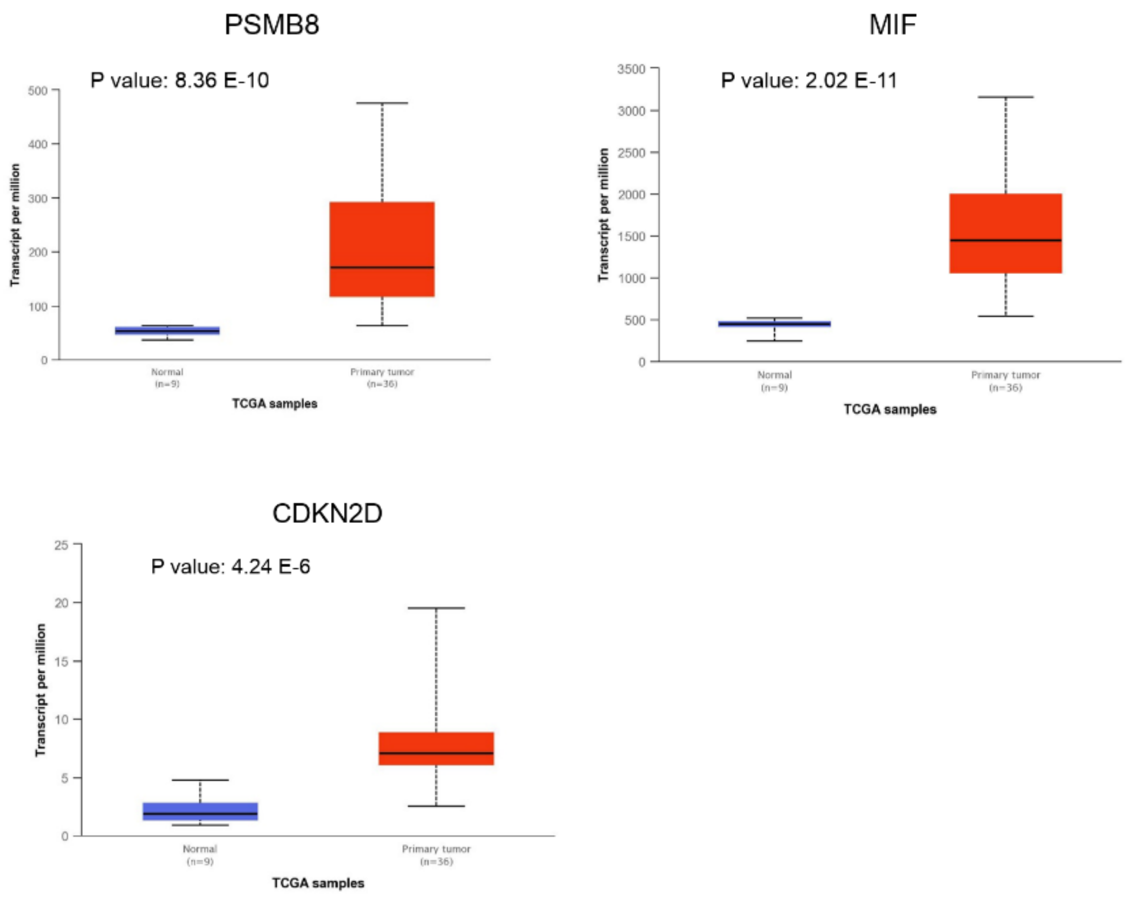

Supplementary Figure S10 The expression levels of PSMB8, MIF, and CDKN2D in cholangiocarcinoma tissues and normal tissue, based on the data of The Cancer Genome Atlas. The levels of these three mRNAs were significantly higher in cancer tissues than in normal tissue (P<0.001).

Supplementary Table S1

Supplementary Table S1. Clinicopathological characteristics of participants in the comprehensive microRNA array analysis

	Gallbladder cancer	Benign gallbladder disease	Healthy Controls
	**GBC**	**Benign**	**HCs**
	**N=3**	**N=3**	**N=10**
**Male, n (%)**	1 (33)	1 (33)	3 (30)
**Age, Median (IQR)**	65 (65-87)	42 (36-78)	73 (57-80)
**Histology, n**	Adenocarcinoma 3	Adenomyomatosis 2<br>Xanthogranulomatous cholecystisis 1<br>Cholesterol polyp 1	N.A.
**Stage (UICC), n**	Ⅳb, 3	N.A.	N.A.
**TNM classification, n**			
N factor	0/1/2 = 0/1/2	N.A.	N.A.
M factor	0/1 = 1/2	N.A.	N.A.
**T-bil (mg/dL)***	0.56 (0.48-0.99)	0.62 (0.55-0.72)	0.62 (0.51-0.80)
**AST (U/L)***	17 (16-51)	19 (19-19)	23 (16-26)
**ALP (U/L)***	289 (84-608)	131 (126-225)	262 (205-487)
**WBC (10^3/μl)***	5.14 (4.06-7.91)	7.46 (6.56-9.28)	5.6 (4.1-6.7)
**CRP (mg/dL)***	1.69 (0.07-3.06)	0.13 (0.09-0.17)	0.12 (0.05-0.25)
**CEA (ng/mL)***	17.9 (2.52-96.77)	1.85 (0.7-4.12)	1.76 (1.1-2.1)
**CA19-9 (U/mL)***	163.3 (13.3-1263)	11.9 (6.7-14.5)	11 (7.3-18.1)

UICC, Union for International Cancer Control

IQR, Interquartile range; N.A., not applicable

* These data are shown as median (range).

Supplementary Table S2

Supplementary Table S2. Diagnostic power of miR-1246, miR-451a, CEA, and CA19-9 in discriminating GBC from Benign and HCs

	AUC	Sensitivity (%)	Specificity (%)	PPV (%)	NPV (%)	Accuracy (%)
miR-1246 in serum EVs	0.646	60.0	66.7	62.5	64.3	63.4
miR-451a in serum EVs	0.664	62.0	75.0	68.9	67.8	68.3
CEA	0.770	60.0	83.3	76.9	69.2	72.1
CA19-9	0.729	58.0	92.6	87.8	70.4	75.9
CEA＋CA19-9＋miR-1246 in serum EVs	0.816	72.0	90.8	87.8	77.8	81.7

AUC, Area Under the Curve; PPV, Positive Predictive Value; NPV, Negative Predictive Value
GBC, Gallbladder cancer; Benign, Benign gallbladder disease; HCs, Healthy controls
EVs, Extracellular vesicles
